# Longitudinal Imaging of Cancer Cell Metastases in Two Preclinical Models: A Correlation of Noninvasive Imaging to Histopathology

**DOI:** 10.1155/2014/102702

**Published:** 2014-03-03

**Authors:** Pavan P. Adiseshaiah, Nimit L. Patel, Lilia V. Ileva, Joseph D. Kalen, Diana C. Haines, Scott E. McNeil

**Affiliations:** ^1^Nanotechnology Characterization Laboratory, Cancer Research Technology Program, Leidos Biomedical Research, Inc., Frederick National Laboratory for Cancer Research, Frederick, MD 21702, USA; ^2^Small Animal Imaging Program, Laboratory Animal Science Program, Leidos Biomedical Research, Inc., Frederick National Laboratory for Cancer Research, Frederick, MD 21702, USA; ^3^Pathology/Histotechnology Laboratory, Laboratory Animal Science Program, Leidos Biomedical Research, Inc., Frederick National Laboratory for Cancer Research, Frederick, MD 21702, USA

## Abstract

Metastatic spread is the leading cause of death from cancer. Early detection of cancer at primary and metastatic sites by noninvasive imaging modalities would be beneficial for both therapeutic intervention and disease management. Noninvasive imaging modalities such as bioluminescence (optical), positron emission tomography (PET)/X-ray computed tomography (CT), and magnetic resonance imaging (MRI) can provide complementary information and accurately measure tumor growth as confirmed by histopathology. *Methods*. We validated two metastatic tumor models, MDA-MD-231-Luc and B16-F10-Luc intravenously injected, and 4T1-Luc cells orthotopically implanted into the mammary fat pad. Longitudinal whole body bioluminescence imaging (BLI) evaluated metastasis, and tumor burden of the melanoma cell line (B16-F10-Luc) was correlated with (PET)/CT and MRI. In addition, *ex vivo* imaging evaluated metastasis in relevant organs and histopathological analysis was used to confirm imaging. *Results*. BLI revealed successful colonization of cancer cells in both metastatic tumor models over a 4-week period. Furthermore, lung metastasis of B16-F10-Luc cells imaged by PET/CT at week four showed a strong correlation (*R* ^2^ = 0.9) with histopathology. The presence and degree of metastasis as determined by imaging correlated (*R* ^2^ = 0.7) well with histopathology findings. *Conclusions*. We validated two metastatic tumor models by longitudinal noninvasive imaging with good histopathology correlation.

## 1. Introduction

Metastasis of cancer cells from primary tumors is one of the leading causes of poor prognosis and death from the disease. Metastatic spread is the root of approximately 90% of cancer-related deaths [1]. Early detection of cancer cell metastasis would be incredibly beneficial for timely therapeutic intervention and management of the disease.

In most preclinical animal studies, gross examination and histopathological analysis are commonly used to evaluate dissemination of cancer cells to secondary sites. These techniques are limited, however, as they cannot be used to adequately monitor tumor development. Furthermore, optical microscopy may have an inadequate field of view (3D morphometric) for thorough tumor evaluation. Noninvasive imaging provides the ability to perform real-time serial imaging to enhance the observation of disease progression and/or evaluate therapeutic treatment response. In addition, noninvasive imaging allows researchers to use fewer animals with greater statistical power.

Orthotopic and metastatic tumor models provide an appropriate tumor microenvironment (seed and soil hypothesis) to mimic tumor growth observed in the clinic [2]. In addition, these models can be appropriate to study complex phenomena such as angiogenesis, metastasis, and invasion of cancer cells [3]. The ability to understand these complex events by noninvasive imaging modalities can help dictate proper therapeutic treatments. A number of orthotopic and metastatic tumor models utilizing the luciferase gene have been developed [4–6]. Bioluminescence imaging (BLI) using orthotopic and metastatic models enables investigators to successfully visualize tumor growth in longitudinal studies and evaluate therapeutic responses at metastatic sites. In addition, the substrate for bioluminescence imaging, D-luciferin, requires oxygen and ATP, thereby directly correlating the presence of viable cells in a tumor to luminescence and decreased luminescence to regions of hypoxia/necrosis and/or effect of chemotherapy within a tumor.

Several novel molecular imaging probes are currently being developed for noninvasive imaging technologies such as positron emission tomography (PET), computed tomography (CT), and magnetic resonance imaging (MRI) to monitor cancer progression and inflammation [7–12]. These imaging modalities have enhanced the clinicians' ability to visualize lesions with improved specificity and sensitivity, intervene with appropriate therapies, and monitor therapeutics' effects noninvasively. In addition to these clinical imaging modalities, bioluminescence and fluorescence imaging are useful methods to monitor tumor growth in primary and metastatic sites in preclinical cancer models. The high sensitivity and stability of luciferase expression in transgene cells (stable over 40 passages) correlate very well with histopathology in xenograft and orthotopic implantation models [13–15]. For example, BLI of liver metastasis of MC38 luciferase-expressing colon cancer cells correlated well with ultrasound imaging [15].

Noninvasive imaging provides an ideal tool to monitor efficacy of chemotherapeutic agents in different tumor models [16–18]. For example, BLI and [^18^F]FDG PET/CT were used to monitor tumor size of human hepatocellular carcinoma cells (HCC-LM3-fluc cells) subcutaneously implanted in Balb/c nude mice, treated with cyclophosphamide or saline vehicle control [16]. In another study, noninvasive BLI of orthotopically implanted gastric cancer cells (SGC7901-Luc) demonstrated decreased signal intensity following docetaxel treatment in comparison to a saline control, correlating therapeutic response analyzed by BLI to tumor weight [19]. In addition, BLI signal strongly correlated with tumor volume measurement in an orthotopic bladder cancer model as determined by MRI using the gadolinium-based contrast agent, Magnevist [13].

In the present study, we validated two metastatic models utilizing noninvasive imaging techniques and correlated the tumor burden using histopathology. The first model (experimental metastasis) utilized two luciferase-expressing cell lines, the human breast cancer MDA-MB-231-Luc and the murine melanoma cancer B16-F10-Luc cells, injected intravenously (i.v.) into the tail-vein. The human breast and murine melanoma cancer cells used in the present study are relevant in evaluating lung metastases, as clinically cells from both types of cancers are known to metastasize to the lung in addition to other organs. Furthermore, the experimental metastasis model provides a quick approach to evaluating tumor growth at metastatic sites and is well defined for several cancer cell lines [5, 20, 21]. In addition to BLI, we employed the radiopharmaceutical [^18^F]FDG PET (glucose analog) to evaluate metabolic activity of the metastatic lesions and T2W-MRI for anatomical tumor location, which are difficult to identify by BLI alone. The second model involved an orthotopic implantation of murine mammary adenocarcinoma cancer cells, 4T1-Luc cells, with longitudinal imaging of tumor progression using whole-body bioluminescence in the presence or absence of an intact primary tumor. The 4T1 cells, a triple negative mammary cancer cell line, were used as cell proliferation and the metastatic potential of this cell line mimics human breast cancer growth in a clinical setting. In addition, we implanted these cells in a syngeneic mouse model to evaluate the role of immune cells during cancer growth, spread, and therapeutic intervention. Results obtained from noninvasive imaging of the 4T1-Luc tumor model correlated to metastatic lesions identified by *ex vivo* imaging of resected organs and by histopathology.

## 2. Methods and Materials

### 2.1. Cancer Cell Lines

Luciferase-expressing cancer cell lines (human breast cancer cells MDA-MB-231-Luc, murine melanoma cancer cells B16-F10- Luc, and murine mammary cancer cells 4T1-Luc) were obtained from Caliper Life Sciences (Hopkinton, MA). Detailed construction of the lentiviral vector system, transfection, and characterization of stable cell lines were previously reported [22, 23]. All three cell lines were cultured in RPMI 1640 medium (HyClone, UT) supplemented with 10% heat inactivated fetal bovine serum (HyClone, UT) without antibiotics and tested negative for human and rodent pathogens prior to initiation of the animal studies. A single cancer cell suspension was prepared for tumor induction in Hanks Balanced Salt Solution with greater than 95% viability as evaluated by trypan blue staining.

### 2.2. Animal Models and Cancer Cell Implantation

Animal care was in accordance with the procedures outlined in the Guide for Care and Use of Laboratory Animals (National Research Council, 1996; National Academy Press, Washington, DC) and animal protocols were approved by the Frederick National Laboratory for Cancer Research Institutional Animal Care and Use Committee. Mouse strains used in the study were female athymic nu/nu (MDA-MB-231-Luc cells), female C57 BL/6 albino (B16-F10-Luc cells), and female Balb/c albino (4T1-Luc cells) (Charles River Laboratory, Frederick, MD), 7–9 weeks of age at procurement, and allowed to acclimate for one week prior to cell implantation. 

#### 2.2.1. Intravenous Experimental Metastatic Model

Female athymic nude (*n* = 5) and C57 BL/6 albino (*n* = 5) mice were physically restrained and intravenously injected into the tail-vein with 2 × 10^6^ MDA-MB-231-Luc cells and 1 × 10^5^ B16-F10-Luc cells, respectively, in 0.1 mL of Hanks Balanced Salt Solution (HBSS) using a 25-gauge needle.

#### 2.2.2. Mammary Fat Pad Spontaneous Metastasis Model

Balb/c mice (*n* = 10) were anesthetized with 3% isoflurane with oxygen carrier gas at 1 L/min flow rate delivered by nosecone before the injection. The site of cancer cell injection was prepped with alcohol followed by Betadine, and a 5 mm incision was made anterior to the rear leg to expose the number 4 inguinal mammary gland fat pad. 4T1-Luc cells were implanted in anesthetized animals 5 × 10^4^ with a total volume of 50 *μ*L HBSS through a 27-gauge needle. Surgically exposing to the fat pad ensured correct injection of cancer cells by visual confirmation of fat pad swelling. A solution of 0.25% bupivacaine was applied to the incision before closing with a sterile wound clip. In a subgroup of animals (*n* = 5), the growth of the primary tumor was measured using calipers and surgically resected when the tumor reached 10 mm in diameter.

#### 2.2.3. Bioluminescence Imaging (BLI)

*In vitro* BLI was performed according to manufacturer's procedures (IVIS SPECTRUM, PerkinElmer Inc., Waltham, MA). Correlation of the BLI signal intensity with the number of cells and optimal imaging time (based on peak luciferin uptake) was determined by imaging varying cell densities (0–5 × 10^5^ cells/well) of MDA-MB-231-Luc and B16-F10-Luc cells using black wall, clear bottom 24 well plates (Costar, Corning, NY). After incubating the cells for 24 h, 400 *μ*L of D-luciferin (30 mg/mL; Goldbio, St. Louis, MO) was added to the cells. A dynamic scan for a maximum of up to 40 minutes with 2 sec exposures was acquired (Emission filter: open, f/stop: 1, binning: medium, 1 scan/minute). Pertinent regions of interest (ROI) were drawn on each well and the BLI output was quantified as total flux (photons/second; Living Image software, version 4.2, PerkinElmer Inc., Waltham, MA). To account for any background luminescence, wells without cells (media alone) or wells with cells but lacking the luciferase substrate D-luciferin were included as controls.

For* in vivo* BLI, animals were injected intraperitoneally with D-luciferin (300 mg/kg [0.05 mL/10 g of body weight]) and anesthetized 5 minutes before the peak luciferin uptake time (determined by the *in vitro* BLI experiment) with 2-3% isoflurane. Isoflurane was reduced to 2% after transferring animals to the imaging chamber. Dorsal and ventral BLI (maximum of five animals) was performed (IVIS, PerkinElmer Inc., Waltham, MA) at the optimal imaging time. Weekly *in vivo* BLI was performed for both models, initiated for the intravenous experimental metastatic model at 3 days after cell implantation and the fat pad spontaneous model was initiated at implantation of cancer cells. Depending on the variability of the bioluminescence signal due to difference in tumor burden, autoexposure (1 second–3 minutes) was used for each cell line/animal. To avoid bleed-over from the implantation site, the primary tumor/injection site was covered using a black glove (Dynarex Corporation, Orangeburg, NY) while imaging metastatic lesions in the thoracic cavity. Moreover, bioluminescence tomography or diffuse light imaging tomography (DLIT) on the metastasis model was performed to quantify the number of cancer cells in the lung by utilizing the filtered 2D bioluminescent sequence (excitation filter: Block, emission filter: 560, 580, 600, 620, and 640 nm) in combination with a surface topography.

Determination of the optimal BLI acquisition for the 4T1-Luc spontaneous metastasis model was assessed by imaging the primary tumors one week after tumor implantation for a 30-minute dynamic scan (emission filter: open, f/stop: 1, binning: medium, 1 scan/minute, exposure time: 0.5–5 second). Metastatic burden was evaluated by drawing a standard rectangular shaped ROI (same size over all time points) over the lung region. In addition, 2D *ex vivo* BLI was performed on a subset of animals (five weeks after tumor implantation). Prior to necropsy, animals were injected intraperitoneally (i.p.) with D-luciferin (300 mg/kg [0.05 mL/10 g of body weight]) and tissues of interest (primary tumor, spleen, liver, lymph nodes, kidneys, femur, heart, brain, adrenal gland, and lung) were resected for *ex vivo* imaging. After *ex vivo* imaging, the excised tissues were fixed in 10% neutral-buffered formalin for histopathological analysis.

### 2.3. 18-Fluorodeoxyglucose-Positron Emission Tomography/Computed Tomography Imaging ([^18^F]FDG-PET/CT)

Whole body [^18^F]FDG PET/CT (Inveon Multimodality, Siemens Medical Solutions USA, Inc., Knoxville, TN) was performed to analyze the anatomical location and metabolic function of the lung metastatic tumor [B16-F10-Luc] model 3 weeks after cell implantation. Animals were fasted but allowed water for 10 hours prior to imaging and i.v. tail-vein-injected with [^18^F]FDG (7.63 ± 1.76 MBq) 60 minutes prior to imaging. Mice were placed in a heated anesthesia (2% isoflurane with oxygen carrier at 1 L/min flow) induction chamber during the 60-minute uptake period to minimize [^18^F]FDG muscle and brown fat uptake. Animal body temperature was maintained before and during imaging sessions using a thermostat controlled circulating warm air imaging table maintained at 37°C. Pulmonary function was monitored during the imaging sessions and the anesthesia was regulated to maintain a pulmonary rate between 50 and 90 bpm. Mice were imaged in the prone position for 5 minutes (CT) followed by 30 minutes (PET) to coregister the metabolic with anatomical image data sets. The following CT acquisition parameters were used: 80 kVp, 500 *μ*A, 200 msec per step, and 120 steps covering 220 degrees. PET list-mode data was acquired using a *γ*-ray energy window of 350–650 keV with a coincidence-timing window of 3.432 ns. CT Cone beam reconstruction resulted in 192 × 192 matrix and PET reconstruction utilized OSEM3D, 4 iterations, resulting in a 256 × 256 matrix. Images were analyzed using ASIPro software, version 6.8.0.0 (Siemens Medical Solutions USA, Knoxville, TN). Coregistration of the data sets was performed according to manufacturer's recommendations for in-line serial CT-PET scanner. Quantification of lung metastatic tumor uptake (%ID) was determined with a manual ROI of the total lung subtracted from a 25% of maximum threshold ROI of the heart. Conversion of counts within an ROI to activity (MBq) was performed according to the manufacturer's procedure.

### 2.4. T2W-MRI

Representative animals from both tumor models were imaged at week 4 using a 3.0 T clinical scanner (Philips Intera Achieva, Best, The Netherlands) with 40 mm diameter solenoid receiver volume coil (Philips Research, Hamburg, Germany). A T2-weighted Turbo Spin Echo (T2W-TSE) sequence was implemented (repetition time (TR) of 3500 ms, echo time (TE) of 65 ms, in plane resolution of 0.19 × 0.19 mm^2^, and slice thickness of 0.5 mm) in the coronal view with respiratory triggering to minimize motion artifacts.

### 2.5. Histopathology

All animals were euthanized by CO_2_ asphyxiation either at the end of the study or due to tumor burden associated clinical signs such as greater than 20% body weight loss, respiratory distress, or paralysis. All mice underwent a thorough necropsy. Tissues were fixed in 10% neutral-buffered formalin, routinely processed, and embedded in paraffin blocks. For both metastasis models, paraffin blocks of all lungs and tissues noted to have abnormalities at the time of necropsy were sectioned at 5 *μ*m, stained with hematoxylin and eosin (H&E), and microscopically evaluated. Stained sections were scanned into digital format via an Aperio ScanScope XT (Leica, Vista, CA). To calculate areas occupied by metastases, the digital images were annotated by a board-certified veterinary pathologist using Aperio ImageScope software (version 11.2.0.780).

#### 2.5.1. Statistical Analysis

Bioluminescence intensity (photons/second) and tumor measurements were expressed as the mean and standard deviation. Regression analysis (Pearson's correlation method) was performed to demonstrate correlation between luminescence signal intensity, area of the lesion, and lung metastatic lesion [^18^F]FDG uptake to histopathology.

## 3. Results

### 3.1. *In Vitro* BLI Validation of Luciferase-Expressing Cancer Cells

**Figure 1:**
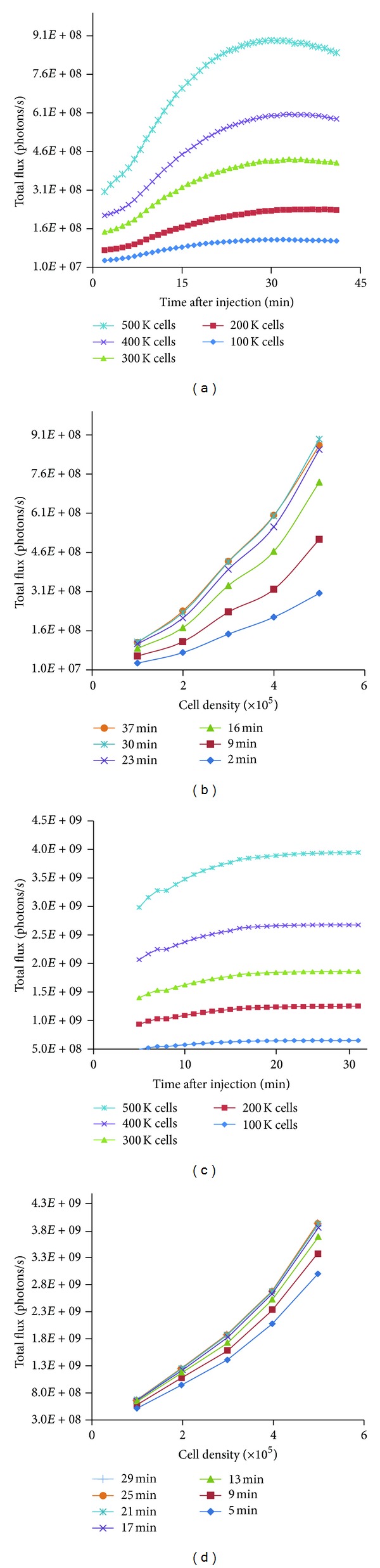
*In vitro *bioluminescence characteristics of B16-F10-Luc and MDA-MB-231-Luc luciferase-expressing cell lines. Murine melanoma B16-F10-Luc ((a) and (b)) and human breast cancer MDA-MB-231-Luc ((c) and (d)) cell lines were serially diluted and imaged with 2 sec exposures for a maximum of up to 40-minute dynamic scan. Kinetic measurement of luciferase activity correlated with the cell density for both cell lines and luminescence activity increased with time. The assay was done in duplicate.

*In vitro* evaluation of BLI was performed to determine the efficiency of the bioluminescent signal with respect to cell density (time-to-peak analysis) and sensitivity of signal detection of the cell lines and to correlate cell density with the *in vivo* signal. Serial dilutions of MDA-MB-231-Luc and B16-F10-Luc cells were plated at different cell densities (0–5 × 10^5^ cells/well) and BLI was performed. An increase in signal was detected with an increase in cell number for both cell lines, suggestive of stability and sustained expression of the luciferase construct (Figure 1). A linear correlation of signal intensity and cell number was obtained for both cell lines in the 3- to 40-minute dynamic range. Based on the dynamic scan, plateau was observed between 22 and 36 minutes, 15 and 30 minutes, and 15 and 22 minutes for B16-F10 cells, MDA-MB-231, and 4T1 cells (data not shown), respectively. These time windows were used as optimal *in vivo* imaging times for the respective cell lines. As previously reported, signal intensity depends on various factors, such as the stable expression of luciferase (signal intensity per cell), signal detection limitation of the instrument, and attenuation of *in vivo* signal by the tissues [20]. All cells were maintained up to 20 passages and assayed for *in vitro* luciferase expression prior to an *in vivo* study.

### 3.2. Validation of Metastasis Models by *In Vivo* BLI

**Figure 2:**
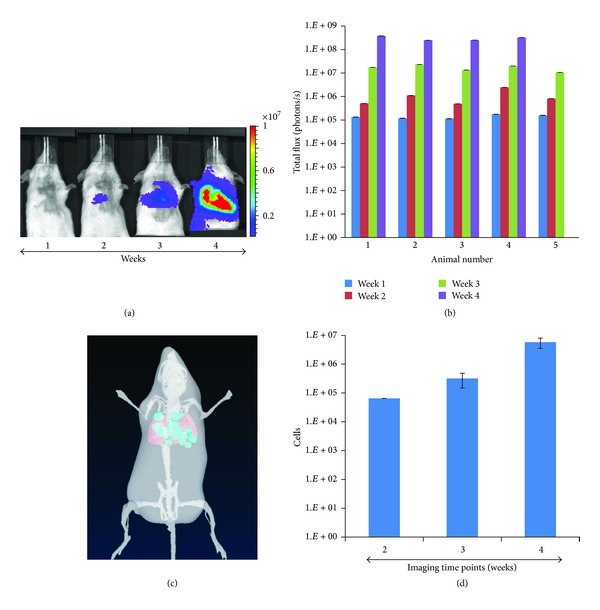
Longitudinal monitoring of tumor growth at metastatic sites by bioluminescence imaging. B16-F10-Luc cells were intravenously injected into female C57 BL∖6 mice (*n* = 5). (a) BLI of a representative animal imaged weekly for 4 weeks. (b) Plot representing quantification of bioluminescence intensity as total flux (photons/second) over 4 weeks for all 5 animals. (c) Three-dimensional reconstruction of DLIT of a representative animal at week 4. (d) Quantification of light intensity bioluminescent cells over 3 weeks from DLIT reconstruction.

Mice were i.v. tail-vein-injected with MDA-MB-231-Luc (2 × 10^6^ cells) and B16-F10-Luc (1 × 10^5^ cells) cells and monitored by BLI weekly to validate the metastasis model. The lung and other organ BLI signals from the MDA-MB-231-Luc cells were comparable to background (see Figure  S1 available online at http://dx.doi.org/10.1155/2014/102702), suggesting rapid clearance of cancer cells from the systemic circulation. The B16-F10-Luc cells successfully colonized the lung as evaluated by increased bioluminescence signal (Figure 2(b)) and histopathological analysis (Figure S2) during the 4-week study. One B16-F10-Luc animal was euthanized 3 weeks after implantation due to excessive tumor burden. In addition, 3D DLIT was performed and demonstrated the presence of luciferase-expressing cancer cells localized around the thoracic region (Figure 2(c)). Bioluminescence quantitation confirmed an increase in signal intensity over the 4-week longitudinal imaging period (Figure 2(d)). DLIT for the MDA-MB-231-Luc model showed relatively fewer cells (0.31% cells at four weeks) in the lung (Figure S1C) suggestive of poor colonization of cancer cells.

### 3.3. Validation of Melanoma Cancer Cell Metastasis by [^18^F]FDG-PET/CT and MRI 

**Figure 3:**
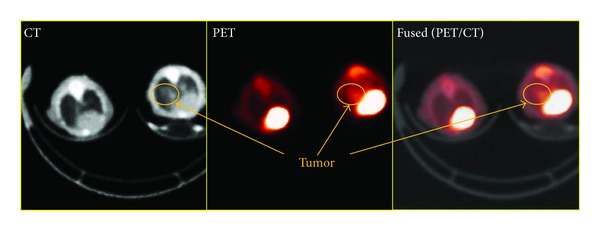
Imaging melanoma cancer cell metastasis by [^18^F]FDG-PET/CT. Evaluation of fused PET/CT images confirms an increase in [^18^F]FDG accumulation in the lungs of animals with metastatic lesions as shown on transverse slices. Images were obtained 21 days after cancer cell injection.

**Figure 4:**
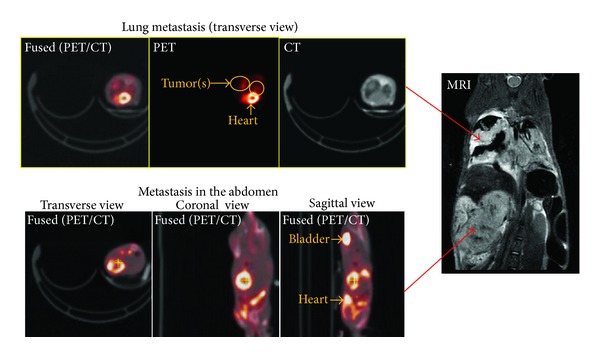
A correlation of [^18^F]FDG-PET/CT and T2W-MRI of melanoma cancer cell metastasis. Accumulation of [^18^F]FDG in the lungs and abdomen as evaluated by fused PET/CT correlated with metastatic lesions observed by T2W-MRI. A representative image from five animals is shown.

**Figure 5:**
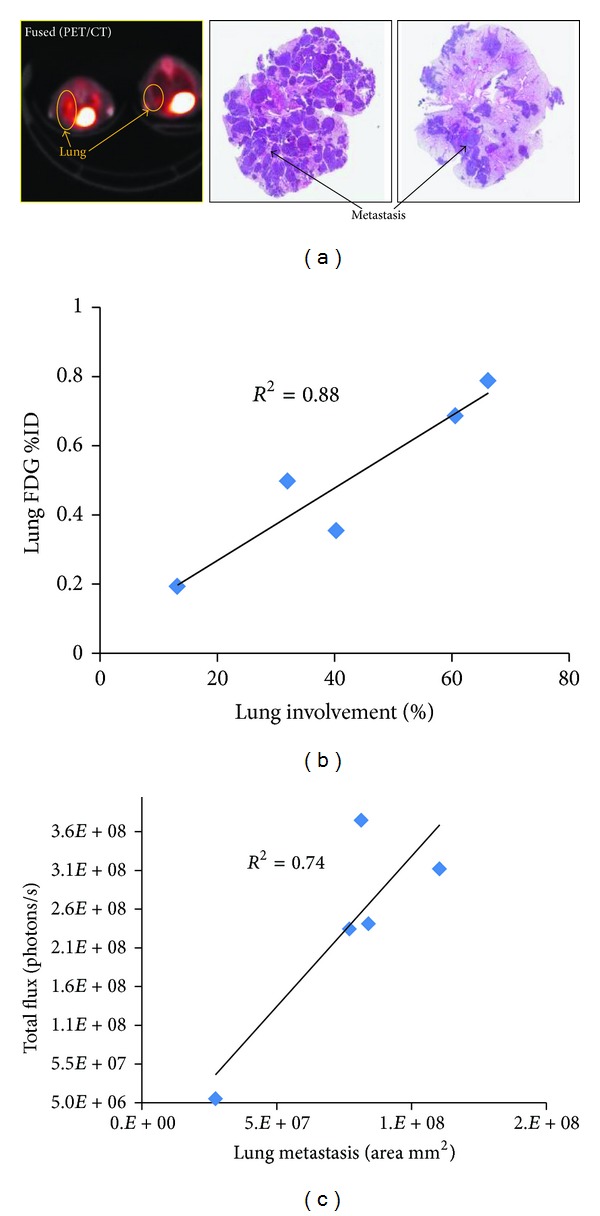
Correlation of [^18^F]FDG-PET imaging of melanoma cancer cell metastasis with histopathology. [^18^F]FDG accumulation in the lungs correlated with the extent and presence of metastasis by histopathology. (a) Representative fused image of PET/CT from 2 animals showing different levels of [^18^F]FDG accumulation. Correlation with tumor nodules in the lung is evident from paraffin section images of the lung stained with H&E. (b) Regression plot showing correlation between lung metastatic lesions identified by [^18^F]FDG-PET imaging and the percent of lung involvement of melanoma cancer cells calculated by histopathology. (c) Regression plot between BLI signal intensity of the thoracic region and the lung metastasis area (mm^2^) as calculated by histopathology. Correlation was calculated using Pearson correlation coefficient analysis.

The metabolic activity of B16-F10-Luc melanoma cells at the metastatic sites was evaluated by the glucose analog PET radiotracer [^18^F]FDG and correlated to anatomical locations by MRI. CT was used to correct the 511 keV photon attenuation and to produce images that provided anatomical landmarks. Accumulation of the [^18^F]FDG was observed in the lung (metastatic lesions) and abdomen and confirmed by coregistration of the PET metabolic image with the anatomical CT (Figure 3) and T2W-MRI (Figure 4). As expected, increased [^18^F]FDG uptake in the heart and bladder was also observed (Figure 4). The uptake of [^18^F]FDG correlated with the extent of tumor cell metastasis as evaluated by the H&E stained section of the lung (Figure 5(a)). By histopathology, there was a metastatic lesion found in the kidney, which was difficult to distinguish from the PET signal due to the renal clearance of the radiotracer (data not shown; Table 1). Regression analysis showed a very good correlation between the percent-injected dose of radiotracer uptake in the lung to percent lung involvement by histopathology and area of lung metastasis to total photon flux (Figures 5(b) and 5(c); Table 1). By histopathology, additional metastatic lesions of melanoma cancer cells were identified in the mediastinum, mediastinal lymph node, and mesentery that did not show accumulation of the radiotracer [^18^F]FDG (Table 1).

### 3.4. Mammary Cancer Metastasis and Correlation with T2W-MRI and *Ex Vivo* BLI

**Figure 6:**
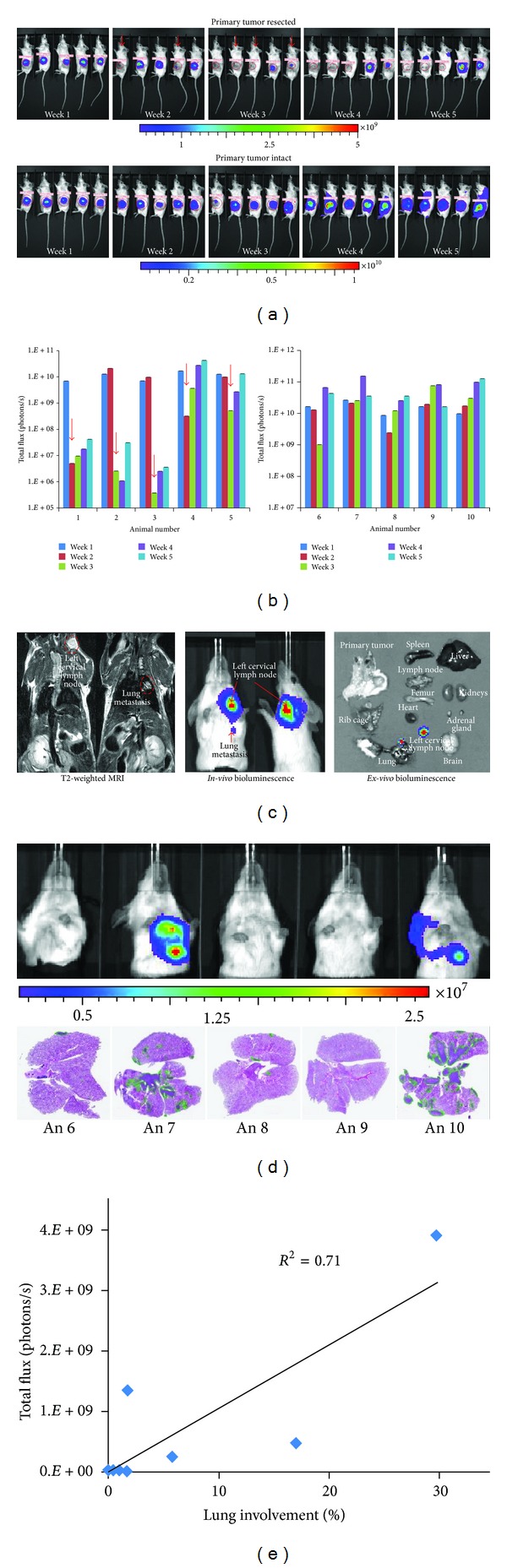
Longitudinal bioluminescence imaging of mammary cancer cell metastasis in a syngeneic mouse model and correlation with histopathology. (a) 4T1-Luc cells, a murine mammary cancer cell line, were orthotopically injected in the mammary fat pad and serial bioluminescence imaging performed 7, 14, 21, 28, and 35 days after cancer cell implantation is shown. Red arrows indicate points at which primary tumors were resected when reaching 10 mm in diameter (top graph). In another group of 5 animals, the primary tumors remained intact (bottom graph). (b) BLI quantification of tumor growth at the implantation site (primary tumor) for both groups; the increase in BLI total signal intensity over time suggests progression of cancer at the primary tumor site. (c) BLI signal intensity at metastatic sites was correlated to T2W-MRI and with *ex vivo* imaging. (d) The correlation of BLI signals with H&E stained lung sections is shown for all animals with intact primary tumors. The green margins around the tumor lesions in H&E stained sections were used to calculate the area or percentage of tumor involvement. (e) The plot shows the correlation between the BLI signals at week 5, with percent tumor involvement in the lung calculated by histopathology.

Longitudinal BLI was performed for the murine mammary cancer cell line (4T1-Luc) orthotopically implanted in the fourth inguinal mammary fat pad with an intact or resected primary tumor (Figure 6(a)). BLI of both animal groups was performed weekly for five weeks after tumor cell implantation. In one group of animals, the primary tumor was resected once it reached 10 mm in diameter, as determined by caliper measurements. Successful tumor growth at the primary site was evident by detection of the BLI signal as early as one week after cell implantation (Figure 6(a)). Metastatic spread of the cancer cells to the thoracic/abdominal regions was observed by weeks 2 and 3 by blocking the BLI signal arising from the primary implantation site (Figure S3). Quantitation of the BL signal over time demonstrated in all groups a progressive increase in cancer cell colonization and tumor growth (Figure 6(b)). Tumor regrowth at the primary site following resection was observed in all animals (Figure 6(b)). In addition, there was no significant difference in survival between animals with intact or resected primary tumors (data not shown). All animals were euthanized by CO_2_ asphyxiation due to neoplasia-related endpoints.

A biphasic growth pattern was observed with an increased tumor cell proliferation during the first three weeks, and regression after the fourth week, which is indicative of the presence of hypoxic regions or central tumor necrosis. As luciferase enzyme activity requires molecular oxygen in addition to ATP to provide a luminescence signal, this could be a limiting factor in determining the difference between hypoxic and necrotic tissues until the tissue is histologically analyzed. The presence of metastatic lesions as identified by BLI showed association with T2W-MRI (as shown with two different slices) at the left cervical lymph node and lung (Figure 6(c)). *Ex vivo* examination by BLI of various organs further confirmed the presence and extent of cancer cell infiltration in the lung and left cervical lymph node.

### 3.5. Correlation of *In Vivo* Imaging with Histopathology 

Histopathological analysis was performed on both metastatic models to determine the presence of tumor cell infiltration in various organs and correlation with BLI. At the end of the study (4 weeks for B16-F10-Luc and 5 weeks for 4T1-Luc cells), animals were euthanized and the presence of lung metastasis was confirmed in all animals (100%) with less frequent metastases noted in other organs. The extent of tumor cells in the lung for both metastatic models varied between animals but showed good correlation (*R* ^2^ = 0.74 for B16-F10-Luc cells and *R* ^2^ = 0.7 for 4T1-Luc cells) with the signal intensity obtained from BLI (Figures 5(c) and 6(e)).

### 3.6. Histopathology Findings

Histopathological analysis identified additional organs (sites) that contained tumor cells in both metastatic models. These findings are summarized in Table 2. Histopathology also confirmed the presence of cancer cells in the adrenal, mediastinum, brain, kidney, liver, thymus, vertebra, and heart for the spontaneous metastatic model with an intact primary tumor. Animals that had the primary tumor resected displayed metastases in the mediastinum, thymus, rib, and adrenal at a lower frequency than the nonresected model.

## 4. Discussion

### 4.1. Noninvasive Imaging of Tumor Growth at Metastatic Sites

The present study provides a method to characterize two syngeneic metastatic models (C57 BL/6 and Balb/c mouse strains) using multiple imaging modalities to detect secondary tumors and validate these findings with histopathology. Both metastatic models were validated using murine cancer cell lines that stably overexpress the luciferase gene at a high level as evaluated by* in vitro* BLI. The BLI signal intensity correlated with the number of cells in an *in vitro* assay demonstrating the ease in monitoring and quantifying proliferation of cancer cells. Tumor growth of B16-F10-Luc cells in the experimental metastasis model and 4T1-Luc cells implanted orthotopically in the mammary fat pad for the spontaneous metastasis model resulted in colonization of cancer cells primarily in the lung. Metastasis observed using syngeneic cancer cells showed similarities to metastasis and dissemination to organs as seen in clinical settings [24]. Immunocompetent animals have an intact immune system that plays a critical role in the progression and metastasis of cancer cells. For instance, tumor growth and metastasis of 4T1 cells in Balb/c animals are influenced by both innate and adaptive immune responses [25].

Longitudinal BLI confirmed the progression of tumor growth at metastatic sites in both tumor models. Similar to a previous report, we observed fewer lung metastases with MDA-MB-231-Luc cells when injected systemically through the lateral tail-vein [22, 26, 27]. Differences in tumor take rate and metastasis can be attributed to the use of different clonal cells and/or mouse strains (e.g., nude and nude-beige mice [22]). Progressive tumor growth was observed with an increase in signal intensity over 4-5 weeks for both tumor models (B16-F10-Luc and 4T1-Luc cells), confirming successful colonization of cancer cells at metastatic sites such as the thoracic region. Using DLIT for the B16-F10-Luc model, we were able to obtain tomographic images of tumor locations based on signal intensity, which was suggestive of lung metastasis. This same technique was employed to reconstruct a 3D distribution of intracranially implanted murine glioma cells in the brain region [19]. In addition to BLI, tumor growth in the lung was confirmed by [^18^F]FDG PET to image metabolic function of the tumor and coregister with anatomical X-ray CT and T2W-MRI. In addition to lung metastasis, correlation of metastasis in the abdomen was observed by [^18^F]FDG PET/CT and T2W-MRI. In an independent study, a similar detection of metastatic lesions in the lung and abdomen was observed with fused CT/PET imaging for the B16 melanoma subline [28]. Similarly, a very high correlation of the mean uptake of PET radiotracer within the lung metastasis was observed in 4 of 5 SCID animals intravenously injected with A375 M-Fluc melanoma cells [29]. Several tumor models have been validated using small animal PET, BLI, MRI, and micro-CT imaging modalities as they provide complementary information [5, 28, 29]. For instance, fusion of small animal PET and micro-CT imaging correlated with metabolic function to the anatomical location of lung tumors [28]. In several reports, systemic administration by tail-vein of different cancer cell lines has resulted in the induction of lung tumors [28, 30], with the exception of the mouse pheochromocytoma luciferase- expressing cell line, which results in liver metastasis [14].

Differences in the route and density of cancer cell injection have resulted in metastatic lesions in various organs [31–33]. For example, intracardiac injection of 10^2^ B16 melanoma cells resulted in bone metastasis, but systemic administration of the same number of cells by i.v. tail-vein did not result in lung metastasis [31]. In contrast, increasing the number of cells to 10^5^ by i.v. tail-vein injection resulted in lung metastasis with no apparent bone lesions. In another study, subcutaneous administration of B16 melanoma cancer cells into the ear pinna resulted in cervical lymph node metastasis with no evidence of distant spread of cancer cells [32]. In addition to the injection route, isolation of cancer cells from a particular organ can significantly bias the dissemination of metastatic lesions to that organ. For example, isolation of brain seeking breast cancer cells (MDA-MB-231 BR) shows preferential metastasis to the brain when cells are injected via an intracardiac route [34]. Similarly, isolation of the MDA-MB-231 D3H2LN subclone showed an increased tumor take rate and spontaneous lymph node metastasis compared to the parental D3H1 subclone cells [22]. Furthermore, there have been discrepancies in the observed metastasis by cancer cells. In one study, intravenous injection of A549-Luc resulted in no detectable metastases [35] while another report showed successful cancer cell colonization in the lung [30]. Differences observed in colonization of cancer cells and detection sensitivity can be attributed to differences in the luciferase construct, the number of cancer cells injected, the injection needle gauge, and other study-related variables.

The tumorigenic potential of cancer cells can also vary depending on the isolation procedure, as observed for colorectal cancer cells, LS174T [33]. Differences in metastatic frequency were observed by intraportal injection with LS174T cells harvested from a culture plate with or without the use of enzymatic trypsin (isolated single cells versus aggregated cells). The presence of intact surface adhesion markers in aggregated cells is important for successful colonization in the liver and is the suggested reason for the difference in metastasis between aggregated and isolated single cells.

### 4.2. Noninvasive Imaging of Metastasis Correlated with Histopathology

Noninvasive BLI monitored the extent of metastasis of cancer cells in both models and correlated with histopathology. The first metastasis model involving an intravenous injection of melanoma cancer cells, B16-F10-Luc cells, demonstrated successful colonization of cancer cells in the thoracic region based on BLI. The presence of metabolic lung metastases was confirmed in all animals (*n* = 5) by coregistration of small animal [^18^F]FDG PET imaging with small animal X-ray CT demonstrating the anatomical and metabolic details of the cancerous cells. Furthermore, the extent of metastasis in the lung strongly correlated with the mean percent of injected dose uptake of the [^18^F]FDG radiotracer in the lung nodules and histology. In addition, T2W-MRI detected lung metastasis as previously confirmed by BLI and PET/CT imaging modalities. Surprisingly, in an independent study the formation of lung metastases by intravenous injection of B16-F10 cells was undetected by MRI, which was evident by PET/CT imaging [28]. Due to the excretion and kinetics of [^18^F]FDG, the observed high [^18^F]FDG uptake in both the kidney and heart made it difficult to differentiate the presence of metastatic lesions from preferential uptake of the radiotracer in these organs, as evidenced by histopathology detection of metastatic lesions in the kidney, in addition to the mediastinum and mediastinum lymph nodes.

In our spontaneous metastasis model using 4T1-Luc cells, we detected metastasis in the thoracic region by BLI and by histopathology in all animals both with and without resection of the primary tumor. The extent of cancer cell infiltration in the lung determined by histopathological analysis correlated with the BLI signal. In addition, lesions identified by BLI correlated with the presence of metastasis in the left cervical lymph node as observed by T2W-MRI and *ex vivo* BLI.

Longitudinal imaging of tumor growth using the BLI technique offers several advantages such as enhanced speed, ease of image acquisition, high throughput, and low cost of operation, making it an attractive noninvasive imaging modality for preclinical models. Histopathology is considered the gold standard in metastases assessment; however, noninvasive imaging provides real-time tumor growth information. BLI has several limitations: signal intensity is dependent on depth (lesions on the surface have higher signal intensity compared to lesions in the deep tissue) and the 2D planar image does not provide the ability to determine lesion depth. Despite these limitations, noninvasive imaging when coupled with histopathology provides a strong correlation for detection of metastatic lesions.

This study provided methods to characterize two metastatic models using multimodal imaging techniques, BLI, PET/CT, and T2W-MRI, with validation of tumor lesions with histopathology. Based on this study, BLI provides a valid noninvasive imaging strategy to monitor tumor growth in longitudinal studies. Using complementary imaging modalities can circumvent inherent limitations of optical imaging such as signal attenuation due to the tissue depth, availability of luciferin substrate, and low levels of ATP and oxygen at the tumor site. Furthermore, application of other imaging modalities and molecular probes such as small animal PET/CT and MRI to image metastatic sites will afford more information about the tumor model (metabolic activity, anatomical location, and tumor volume) and can assist in employing relevant therapeutic intervention strategies. Further validation of metastatic sites with histopathological analysis alongside noninvasive imaging modalities will be helpful in evaluating tumor growth and effectiveness of therapy.

## 5. Conclusion

Overall, we were able to validate two syngeneic metastatic models using various imaging technologies to understand the tumor growth profiles at secondary sites noninvasively and longitudinally. BLI provides a sensitive technique for imaging the whole body, for detection of metastatic lesions, and correlation with T2W-MRI and histopathological analysis. Metabolic [^18^F]FDG PET/CT of the B16-F10 metastatic model further confirmed the presence of tumors detected by BLI. Each of the imaging modalities provides a specific advantage: small animal PET provides metabolic activity; micro-CT contributes anatomical information of the lung; and T2W-MRI provides morphological and tumor volume data. Based on the present study, it is advantageous to utilize complementary imaging modalities with correlation to histopathology to maximize the understanding of a tumor model and/or response to a therapeutic treatment.

## Figures and Tables

**Table 1:** Summary of melanoma cancer cell metastases detected by histopathology and [^18^F]FDG-PET.

		Mouse number				
Tissues with melanoma cancer cell metastasis	Method of detection	1	2	3	4	5
Lung	[^18^F]FDG-PET	+	+	+	+	+
	Histopathology	+	+	+	+	+
Mediastinum	[^18^F]FDG-PET	−	−	−	−	−
	Histopathology	+	−	−	−	+
Mediastinal lymph node	[^18^F]FDG-PET	−	−	−	−	−
	Histopathology	−	−	+	+	−
Kidney*	[^18^F]FDG-PET	+	+	+	+	+
	Histopathology	−	+	−	−	+
Mesentery	[^18^F]FDG-PET	−	−	−	−	−
	Histopathology	−	−	−	−	+
Percentage of lung section involved in melanoma cancer cell metastasis		40.3	13.2	32.0	66.2	60.6
Percent injected dose of [^18^F]FDG in the lung		0.35	0.19	0.49	0.78	0.69

+: metastasis; −: no metastasis; *[^18^F]FDG-PET is cleared primarily through kidney and may not be a true reflection of presence of metastasis.

**Table 2:** Comparison of organs with tumor lesions detected by histopathology between the two metastatic models.

Organs	Experimental metastasis (tail-vein injection)	Spontaneous metastasis (mammary fat pad injection)	
	B16-F10	4T1 (primary tumor resected)	4T1 (primary tumor intact)
Lung	10/10 (100%)	5/5 (100%)	5/5 (100%)
Mediastinal lymph node	4/10 (40%)	—	—
Mediastinum	5/10 (50%)	2/5 (40%)	3/5 (60%)
Vertebra/spinal cord	2/10 (20%)	—	1/5 (20%)
Brain	—	—	2/5 (40%)
Kidney	2/10 (20%)	—	1/5 (20%)
Liver	—	—	2/5 (40%)
Thymus	—	2/5 (40%)	2/5 (40%)
Rib	—	2/5 (40%)	—
Adrenal	—	1/5 (20%)	4/5 (80%)
Heart	—	—	1/5 (20%)
Mesentery	1/10 (10%)	—	—
Subcutaneous lymph node	—	2/5 (40%)	1/5 (20%)
Sternum and foreleg	—	—	1/5 (20%)
